# A Novel Tool to Measure Extracellular Glutamate in the Zebrafish Nervous System *In Vivo*

**DOI:** 10.1089/zeb.2016.1385

**Published:** 2017-06-01

**Authors:** Ryan B. MacDonald, Nachiket D. Kashikar, Leon Lagnado, William A. Harris

**Affiliations:** ^1^ Department of Infection, Immunity and Cardiovascular Disease, Medical School and the Bateson Centre, University of Sheffield, Sheffield, United Kingdom.; ^2^ Department of Physiology, Development and Neuroscience, University of Cambridge, Cambridge, United Kingdom.; ^3^ Sussex Neuroscience, School of Life Sciences, University of Sussex, Brighton, United Kingdom.

**Keywords:** glia, retina, glutamate, nervous system

## Abstract

Glutamate is the major excitatory neurotransmitter in the brain. Its release and eventual recycling are key to rapid sustained neural activity. We have paired the *gfap* promoter region with the glutamate reporter molecule, iGluSnFR, to drive expression in glial cells throughout the nervous system. *Tg(gfap:iGluSnFR)* is expressed on the glial membrane of Müller glia cells in the retina, which rapidly respond to stimulation and the release of extracellular glutamate. As glial cells are associated with most, if not all, synapses, *Tg(gfap:iGluSnFR)* is a novel and exciting tool to measure neuronal activity and extracellular glutamate dynamics in many regions of the nervous system.

Glutamate is the major excitatory neurotransmitter in the brain. Its release and eventual recycling are key to rapid sustained neural activity.^1^ Glial cells play a key role in the uptake and recycling of glutamate from the synaptic cleft. iGluSnFR has been used to study synaptic activity by measuring glutamate dynamics in the zebrafish nervous system.^2,3^ Previous work has also used iGluSnFR in glial cells; however, this was done transiently in the mouse using viral vectors.^2,4^ As such, we designed a transgene to stably express iGluSnFR in the glial cells of the zebrafish nervous system. We report a novel transgenic zebrafish, *Tg(gfap:iGluSnFR)*, that displays the glutamate-sensitive fluorescent reporter iGluSnFR specifically on the membrane of glial cells (Figure 1A–C). This molecule is expressed on the glial membrane in many brain regions and rapidly responds to stimulation and the release of extracellular glutamate (Figure 1D–F, Supplementary Data; Supplementary Data are available online at www.liebertpub.com/zeb). Thus, pairing the sensitivity of iGluSnFR and optical transparency of the zebrafish provides a powerful tool for understanding glutamate dynamics in neural tissues *in vivo*.

**FIG. 1.**
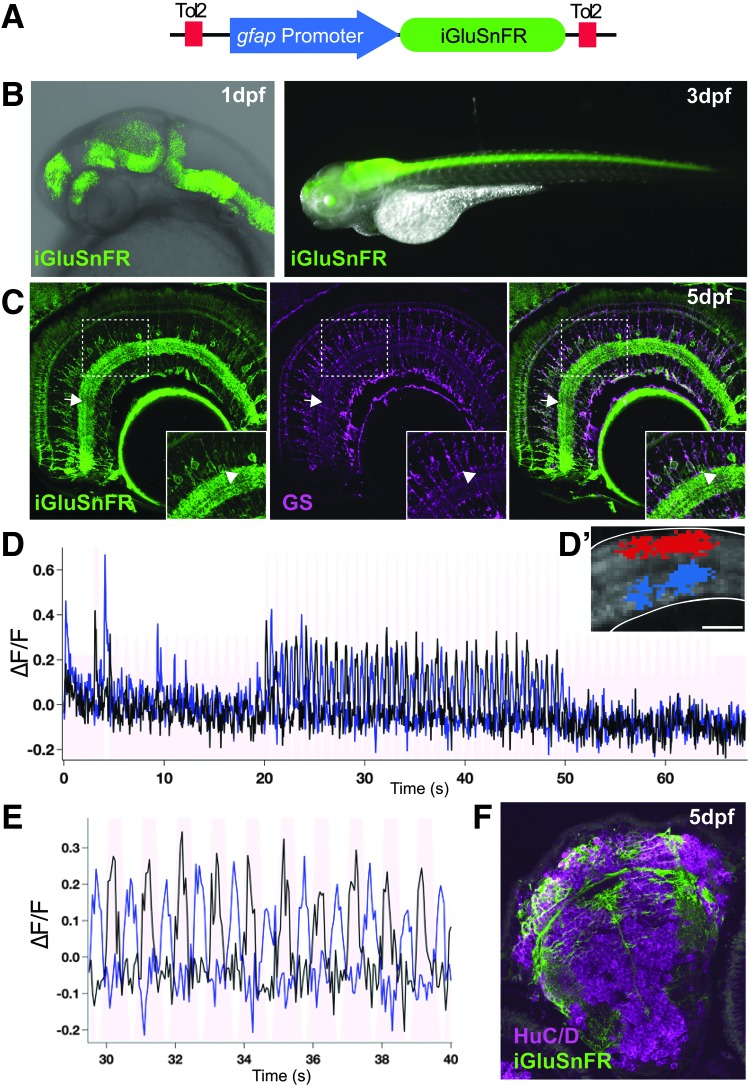
**(A)** To express iGluSnFR specifically in glial cells, we used the *gfap* promoter region to generate the *Tg(gfap:iGluSnFR)* transgenic line. **(B)** Whole mount fluorescent images of the *Tg(gfap:iGluSnFR)* embryos showing expression begins at 1 day postfertilization (dpf) in the embryonic and in the maturing nervous system at 3 dpf. **(C)** Sections showing iGluSnFR expression in the retinal Müller glia cells, labeled with the glutamine synthetase (GS) antibody, up to at least 5 dpf. *Inset* shows that all GS-positive cells are also iGluSnFR positive in the retina. The iGluSnFR signal is enriched in the inner plexiform layer (*arrow*) presumably because of the presence of low-level extracellular glutamate in the synaptic layer of the retina. There are occasional GS-negative cells, presumably neurons, expressing iGluSnFR in the inner nuclear layer of the retina (*arrowhead*). These cells may be labeled because of early activity of the *gfap* promoter in progenitors during the genesis of neurons, which has been noted previously.^5^ **(D)** iGluSnFR in the retina responds to light stimulation as characterized by activity-dependent changes in fluorescence over time in the ON (*black plot*) and OFF (*blue plot*) regions of the inner plexiform layer (*arrow*). (**D**’) An example of the region of interest used to measure ON (*blue*) and OFF (*red*) responses in the inner plexiform layer of the retina. Scale bar denotes 10 μm. **(E)** Zoom of D between 30 and 40 s clearly shows the light-dependent responses in the retina. **(F)** iGluSnFR is also expressed in the glial cells and neuropil of the brain, but not the neurons (HuC/D positive) in sections at 5 dpf.

## Supplementary Material

Supplemental data
