# Supplementary material for: A Novel Tool to Measure Extracellular Glutamate in the Zebrafish Nervous System In Vivo
Source: Zebrafish. 2017 Jun 1;14(3):284–6. doi: 10.1089/zeb.2016.1385 (PMC5466054; doi:10.1089/zeb.2016.1385)
Supplement: Supplemental data [file Supp_Data.pdf]

Supplementary Data

Fish Maintenance

Zebrafish were maintained and bred at 26.5°C. All animal work was approved by the Local Ethical Review Committee at the University of Cambridge and performed according to the protocols of project license PPL 80/2198 and according to institutional guidelines of the University of Sussex Ethical Review Committee.

DNA Constructs and Generation of Transgenic Fish

For the generation of the *gfap*:iGluSnFR construct, we modified the *gfap*:dTomato Tol2 construct.[1] The *GFAP:d-Tomato* construct contains 7.3 kb of the 5′ upstream sequence of zebrafish *gfap* gene[2] in the pT2KXIGDin plasmid.[3] A 1.8kb iGluSnFR gene from the pCMV.iGluSnFR plasmid (Addgene: 41732) containing the coding sequence using primers; 5′-CTCGAGTGATCCCGCCACCATGGAGACA GACACACTCCTG-3′ and 5′-GCTAGCCGCCTAACGTG GCTTCTTCTG-3′ was cut using Xho1 and Nhe1 to replace dTomato and make *gfap*:iGluSnFR plasmid.

Microinjection of the *gfap*:iGluSnFr plasmid DNA was injected with Tol2 transposase mRNA as has been previously described.[4] Three stable transgenic lines were subsequently identified using fluorescence in the spinal cord and retina. All three transgenic zebrafish were identical in expression pattern, as such we used a single line for our analysis named *Tg(gfap:iGluSnFr)*[3313].

Antibody Staining and Imaging

Whole mount imaging was carried out using the Leica MZ10 F stereomicroscope (Leica Microsystems) and the Leica DFC3000 G monochrome camera. Laser scanning confocal imaging was performed using an Olympus FV1000 microscope (Olympus) with a 60× silicone oil immersion objective (1.3 NA) using the FV10-ASW software (Version 4.2; Olympus), or cryosections with an SP2 microscope (Leica Microsystems) with a 63× (1.2 NA) water immersion objective using the LCS Leica Confocal Software (Leica Microsystems). Image analysis was performed using ImageJ or Volocity Software (Perkin Elmer).

Immunostaining was performed using alexa-conjugated secondary antibodies (Invitrogen) and the following primary antibodies: mouse antiglutamine synthase (1:150, mab302; Millipore), rabbit anti-GFP (1:200, ab290; Abcam), and mouse anti-HUC/D (1:200, 16A11; Invitrogen). Cryosections were taken at 12 μm thickness using a Jung Frigocut cryostat (Leica).

Stimulation and Recording of iGluSnFR Fluorescence

The sine contrast waveforms for visual stimuli were generated on a Mac mini computer using Igor (Wavemetrics) running mafPC (courtesy of M.A. Xu-Friedman; www.biology.nsm.buffalo.edu/Xu_Friedman/mafPC/sign_in.html). To generate light stimuli, the waveforms were engendered with an amber LED (M590L3—Amber [590 nm] mounted LED; Thorlabs). For *in vivo* recordings, zebrafish larvae were embedded into 3% low melting point agarose and mounted on a glass slide (Biogene, Kimbolton, Cambridgeshire, United Kingdom). The glass slide was housed in a tank containing E2 buffer[5] with three black walls and one screen made of photofilm such that one eye was facing the screen. To prevent eye movement, ocular muscles were paralyzed by nanoliter injections of α-bungarotoxin (2 mg/mL) behind the eye. The mean irradiance at the screen was 12.68 nW mm^{-2}. Around the mean irradiance, we applied first a positive and negative step followed by 1 Hz sine wave—10% for 15 s, 90% for 30 s, and finally 10% for 15 s. Visual stimulation was synchronized with image acquisition using the data acquisition interface ITC-18 (HEKA Electronik; Lambrecht/Pfalz, Germany). Image acquisition and laser scanning were controlled on a separate Windows PC using ScanImage.[6] Imaging was carried out using a Scientifica two-photon microscope equipped with a mode-locked titanium–sapphire laser (Chameleon, Coherent) tuned to 915 nm. Emission was passed through GFP emission filters (HQ 535/50; Chroma Technology) before detection with photomultiplier tubes (Hamamatsu).

References

1. Satou C, Kimura Y, Higashijima S. Generation of multiple classes of V0 neurons in zebrafish spinal cord: progenitor heterogeneity and temporal control of neuronal diversity. J Neurosci 2012;32:1771–1783.
2. Bernardos RL, Raymond PA. GFAP transgenic zebrafish. Gene Expr Patterns 2006;6:1007–1013.
3. Urasaki A, Morvan G, Kawakami K. Functional dissection of the Tol2 transposable element identified the minimal cis-sequence and a highly repetitive sequence in the subterminal region essential for transposition. Genetics 2006;174:639–649.
4. Kawakami K, Shima A, Kawakami N. Identification of a functional transposase of the *Tol2* element, an *Ac*-like element from the Japanese medaka fish, and its transposition in the zebrafish germ lineage. Proc Natl Acad Sci U S A 2000;97:11403–11408.
5. Westerfield M: *The Zebrafish Book. A Guide for the Laboratory use of Zebrafish (Danio Rerio).* University of Oregon Press, Eugene, Oregon, 2000.
6. Pologruto TA, Sabatini BL, Svoboda K. ScanImage: flexible software for operating laser scanning microscopes. Biomed Eng Online 2003;2:13.
